# Type 2 Diabetes Mellitus: A Review of Multi-Target Drugs

**DOI:** 10.3390/molecules25081987

**Published:** 2020-04-23

**Authors:** Angelica Artasensi, Alessandro Pedretti, Giulio Vistoli, Laura Fumagalli

**Affiliations:** Dipartimento di Scienze Farmaceutiche, University Degli Studi di Milano, 20133 Milano, Italy; angelica.artasensi@unimi.it (A.A.); alessandro.pedretti@unimi.it (A.P.); giulio.vistoli@unimi.it (G.V.)

**Keywords:** diabetes mellitus, type 2 diabetes mellitus, multi-target compounds, multi-target drugs

## Abstract

Diabetes Mellitus (DM) is a multi-factorial chronic health condition that affects a large part of population and according to the World Health Organization (WHO) the number of adults living with diabetes is expected to increase. Since type 2 diabetes mellitus (T2DM) is suffered by the majority of diabetic patients (around 90–95%) and often the mono-target therapy fails in managing blood glucose levels and the other comorbidities, this review focuses on the potential drugs acting on multi-targets involved in the treatment of this type of diabetes. In particular, the review considers the main systems directly involved in T2DM or involved in diabetes comorbidities. Agonists acting on incretin, glucagon systems, as well as on peroxisome proliferation activated receptors are considered. Inhibitors which target either aldose reductase and tyrosine phosphatase 1B or sodium glucose transporters 1 and 2 are taken into account. Moreover, with a view at the multi-target approaches for T2DM some phytocomplexes are also discussed.

## 1. Introduction

Diabetes Mellitus (DM) is a multi-factorial chronic health condition triggered by several genetic and/or environmental factors [1,2]. Indeed, this pathology is characterized by strong familiarity and the frequency of diabetes varies in different ethnicities, such as black and Hispanic people, and some minorities, like American Indians and Natives of Alaska, are more likely to have diabetes for a specific genetic profile.

The World Health Organization (WHO) Global report on diabetes shows that the number of adults living with diabetes has almost quadrupled since 1980 to 422 million adults [3] and is expected to increase to 693 million by 2045 [4]. The disease is characterized by high blood sugar levels, due to a deficiency of concentration and/or of activity of insulin, the pancreatic hormone involved in managing glycaemia.

There is no cure for diabetes so far, but it can be treated and controlled. Pharmacological therapy and/or insulin may be required in order to maintain the blood glucose level as near as possible to normal and to delay or possibly to prevent the development of diabetes-related health problems. However, disease management can be helped also by healthy eating and physical exercise.

For determining the right therapy, the involved type of diabetes plays a key role and in 2018 American Diabetes Association (ADA) proposed the following classification [5]:

1. Type 1 diabetes mellitus (T1DM): due to autoimmune β-cell destruction, usually leading to absolute insulin deficiency;
2. Type 2 diabetes mellitus (T2DM): due to a progressive loss of β-cell insulin secretion frequently on the background of insulin resistance;
3. Gestational diabetes mellitus (GDM): diabetes diagnosed in the second or third trimester of pregnancy that was not clearly overt prior to gestation;
4. Specific types of diabetes due to other causes, e.g., monogenic diabetes syndromes (such as neonatal diabetes and maturity-onset diabetes of the young (MODY)), diseases of the exocrine pancreas (such as cystic fibrosis and pancreatitis), and drug- or chemical-induced diabetes (such as with glucocorticoid use, in the treatment of HIV/AIDS, or after organ transplantation).

Since T2DM is suffered by the majority of diabetic patients (around 90–95%) this review focuses on the potential drugs acting on multi-targets involved in the treatment of this type of diabetes.

### 1.1. Type 2 Diabetes Mellitus (T2DM)

Type 2 Diabetes Mellitus (T2DM) has been referred for long time as non-insulin dependent diabetes, or adult-onset diabetes characterized by insulin resistance, which could progressively worsen to absolute resistance, but in the past decade reduced β-cell function has been recognized as a key problem in T2DM [6].

Indeed, in the past two decades, T2DM emerged as a new and very serious health problem also in children [7,8]. The studies carried out on children demonstrated the co-existence of obesity, insulin resistance, and β-cell dysfunction as observed in older T2DM patients [9].

**Figure 1:**
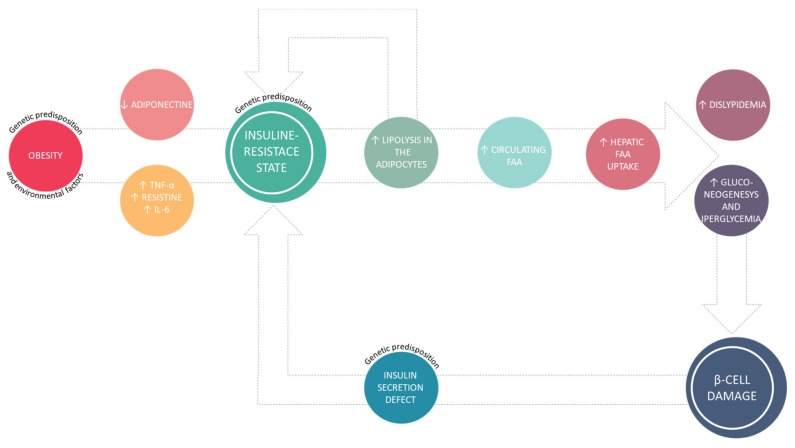
Pathophysiology of type 2 diabetes mellitus (T2DM).

This association can be appreciated in Figure 1.

The adipose tissue is an endocrine organ that can secrete several hormones and cytokines [10], namely TNF-α, IL-6, resistin, which are able to induce a chronic inflammation state and insulin-resistance. Furthermore, in obese patients with metabolic syndrome is more common to observe low adiponectin levels [11] and a leptin-resistance state [12]. Leptin is a hormone with orexigenic activity, that helps to regulate energy balance by inhibiting hunger [13], while adiponectin is a peptide synthetized by adipocytes that exhibits anti-inflammatory, anti-atherogenic effects, and it also is an insulin sensitizer [14]. This metabolic dysfunction leads to insulin-resistance with consequences primarily on adipose, muscular, and hepatic tissues [15]. In this situation, insulin has no anti-lipolytic effect, consequently there is an increased production and secretion, in the systemic circulation, of fatty free acid (FFA) [16] that also are responsible for the insulin-resistance state. Elevated plasma FFA concentrations are associated with increased liver production and secretion of glucose [17]. Moreover, cholesterol and triglycerides concentrations also are increased, especially LDL, [18] with a negative impact on the cardiovascular system [19].

Furthermore, thanks to genome-wide investigations (GWAS) many genes that increase T2DM risk have been also identified and some genes are believed to be important for β-cell function, β-cell development, or the regulation of β-cell mass [20,21]. These findings have supported the relative contributions between insulin resistance and β-cell dysfunction to the pathophysiology of T2DM [22].

Usually, the early stage of this pathology could be asymptomatic, or symptoms could be so mild that they go unnoticed. Therefore, it could be remained undiagnosed for many years, thus rendering truly difficult an accurate estimation of the people suffering from the disease. When present, symptoms include frequent urination, excessive thirst and hunger, fatigue, blurry vision, slow-healing wounds and tingling, pain, or numbness in the hands/feet. Since diabetes could be asymptomatic for a long period of time, T2DM’s diagnosis often coincides with a concomitant disease. When symptoms are missing, diagnosis is made when Fasting Plasma Glucose (FPG) is greater than or equal to 126 mg/dL or when Oral Glucose Tolerance Test (also called the OGTT) is greater than or equal to 200 mg/dL or when HbA_1c_ (i.e., glycated hemoglobin) is greater than or equal to 48 mmol/mol (6.5%). When the symptoms are present, diabetes is diagnosed when blood glucose is greater than or equal to 200 mg/dL. A significant role in the pathology is played by the chronic hyperglycemic exposition to tissues, and especially to blood vessels, that increase the risk of development of comorbidities, namely micro and macrovascular complications. The former include retinopathy, nephropathy, and neuropathy. The latter, which can be often found in patients with longstanding diabetes, include stroke, congestive heart failure, coronary heart disease, myocardial infarction, and peripheral vascular disease.

All of these complications result in a reduction in life expectancy by even ten years among diabetic patients.

Furthermore, several risk factors are associated with T2DM:

- Genetic influences
  Genetics play a very strong role in the development of T2DM [23]. The most common forms of T2DM are polygenetic, so there are changes in multiple genes, but there are also some rare forms of diabetes that are caused by single gene mutation, known as monogenic diabetes. It is important not be confused with T1DM, so it is crucial to have a correct diagnose in order to receive a proper treatment.
- Environmental influences
  New understandings of the progression of T2DM are related both to the lifestyle and to the gut microbiota and the dynamics that leads to microbiome dysbiosis [24]. This change in the microbiota composition is able to reshape intestinal barrier functions and to induce metabolomic and signaling pathways related to the insulin resistance.
- Age
  Until two decades ago, T2DM was usually found in adults and seniors. This was consequential to the increase of insulin resistance due to body composition modification (less muscle in favor of more adipose tissues), to the reduced “sugar burning” capacity and to the progressive decrease of physical activity.
  Leading to the impressive growth of obesity rate, the average age of onset is lowering, and diabetes can now be found even in children.
- Obesity
  A person with a body mass index (BMI) equal to or greater than 30 kg/m^2^ is generally considered obese [25,26].
  The increase of adipose tissue is a primary risk factor in diabetes. In fact, there is a direct correlation between fat percentage and insulin resistant cells, especially if the fat is concentrated in the abdominal area.
  Around 80% of T2DM patients are obese, which is not, however, a necessary condition to develop diabetes.
- Poor physical fitness
  Sedentary lifestyle may increase risk of T2DM [27,28]. Physical activities help to control body weight and lower blood glucose in addition to many other benefits.
- Hypertension and high triglycerides levels
  These are conditions usually associated with insulin resistance, so they increase diabetes risk.
- Smoking
  Smoking is associated with diabetes and other health conditions such as cancer and heart diseases.
- Gestational Diabetes
  Women, that develop diabetes during the pregnancy, have higher risk of suffering for T2DM later in life.
- Polycystic Ovary Syndrome
  Polycystic Ovary Syndrome (PCOS) is a common hormonal disorder that causes irregular menstrual cycles, hirsutism, acne, and, frequently, obesity.

Intervening on modifiable risk factors can be extremely efficacious: by taking proactive changes the risk for diabetes can decrease or delay its progression, in addition of improving the overall life quality.

The treatment of patients with T2DM is challenging, since there is no cure available at the moment, but glycaemia can be controlled by pharmacological therapy. Furthermore, a reliable glycemic control is not the only goal of a proper diabetic management, and other important objectives to be pursued should include reducing the body weight as well as alleviating the symptoms and preventing micro and macrovascular damage.

There are many anti-diabetic drugs that exert clinical effects via different mechanisms. The four major groups of anti-diabetic agents are:

a) biguanides, like metformin, which reduce gluconeogenesis in the liver;
b) insulin secretagogues which stimulate the pancreas to secrete insulin and include drugs such as sulfonylureas;
c) insulin sensitizers which improve sensitivity of peripheral tissues to insulin and include thialzolidinediones and;
d) insulin or its analogues which provide insulin exogenously in the form of recombinant insulin.

Metformin is the first-line pharmacotherapy for T2DM. Besides reducing the glucose level, this has an insulin-sensitizing effect with multiple actions on tissues such as the liver, skeletal muscle, endothelium, adipose tissue, and the ovary. Only if after three months the levels of HbA_1c_ is higher than 7.0%, a second medication can be added. Unluckily, metformin has several side effects (from mild to serious) that cause lack of adherence and therefore it is the antidiabetic oral therapy with the lowest compliance [29].

**Figure 2:**
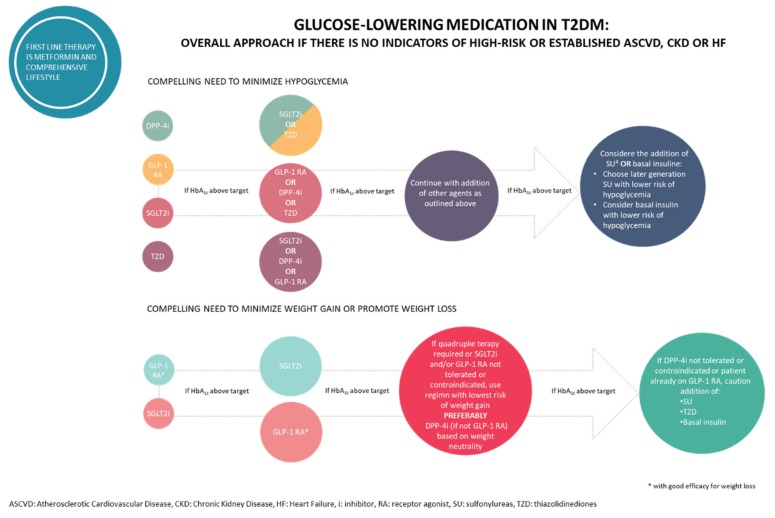
Glucose-lowering medication in T2DM.

Although the existence of plenty of diabetic drugs, other drug monotherapy proved unsuccessful in providing satisfactory managing blood glucose levels and the other comorbidities and therefore therapeutic management is often achieved by combinations therapy with drugs that act with different mechanism of actions as illustrated in Figure 2 [30]. However, this strategy may be affected by problems related to the polypharmacological approach, such as several side effects, toxicity and unwanted drug–drug interactions. Nevertheless, the benefit of these combinational therapies is often compromised by low patient compliance, which could be improved by the development of two or more active components co-formulated in a single tablet.

An alternative strategy in order to combine all these needs is a single molecule that selectively modulates different targets and may have potential for an improved balance of efficacy and safety compared to single-target agents.

### 1.2. Multi-Target Compounds

Traditionally drug design had the aim of targeting selectively a single biological entity in order to avoid other interactions that could potentially lead to unwanted side effects. However, this approach is now considered outdated and over the past years most efforts in drug design were made to develop compounds that are able to exert numerous physiological actions especially for diseases of complex etiology, such as cancer, inflammation, central nervous system (CNS) disorders, and diabetes.

The design of this multi-target ligands must focus on the selection of suitable targets, which have to be well characterized and preferably implicated in different pathways of the disease, and on the optimization of the relative potency of the compound towards each receptor.

Screening and knowledge-based approaches represent the classical way to design multi-target ligands, nevertheless fragment-based and in silico screening have been proposed as a potentially attractive route for the multi target drug design process.

**Figure 3:**
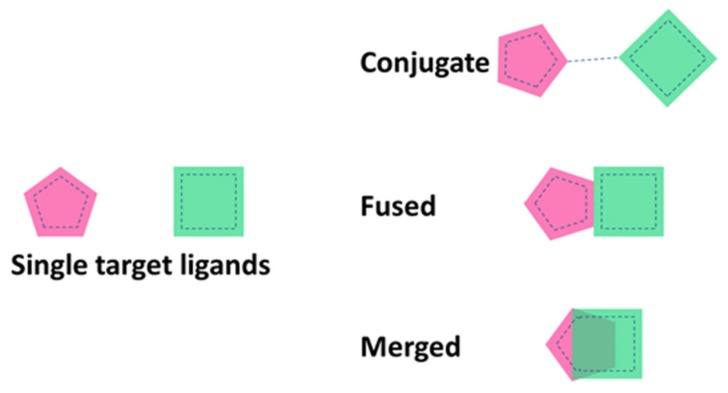
Different strategies to design multi-target ligands.

The knowledge-based approach also known as “framework combination” relies upon structure–activity relationship (SAR) knowledge of every single target involved, in which the pharmacophores are combined in different ways (as represented in Figure 3), where they can be connected by a linker, or they can be overlapping in the same compound or highly integrated in a multi-target drug [31].

Another way is to screen sets of compounds against several targets. A modern approach is the high-throughput screening (HTS), which allows large, diverse compound sets to be screened against several targets of interest, in parallel.

Finally, *in silico* methods gained an increasingly popularity as multi-target drug design tools. A promising tool is the fragment based drug design (FBDD) approach where fragments, even when binding weakly to the biological target, are identified. These fragments are then expanded or linked together to produce drug leads with a high affinity.

Other well-known *in silico* methods, such as molecular docking, pharmacophore analysis, quantitative structure activity relationship (QSAR), machine learning, and their various combinations, have been extensively used.

Though the multi-target approach has only been purposely applied in the last decades, many of the previously known therapeutic agents are in fact multi-target ligands [32], which is especially true for those drugs that were discovered by serendipity, phenotypic screening, or traditional medicine.

Nevertheless, in the state of metabolic disturbance, several major enzymes are abnormally expressed, and they could be interesting targets in drug development. Hence, again, multimodal drugs, which could reduce hyperglycemia and concomitantly inhibit the progression of complications, may offer a valuable therapeutic option.

## 2. Incretin-Based Therapies

### 2.1. Overview

Enhancing the incretin effects is a prominent approach to successfully treat diabetes and to control obesity.

**Figure 4:**
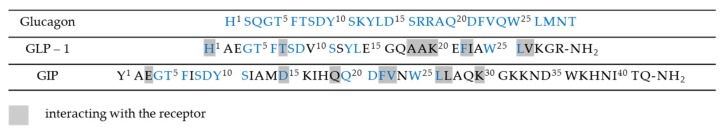
Amino acid sequences of glucagon-like peptide 1 (GLP-1), glucagon, and of glucose-dependent insulinotropic polypeptide (GIP).

Incretin-based therapies exploit the actions of the glucose dependent insulinotropic polypeptide hormone (GIP) and the glucagon like peptide 1 (GLP-1) [33], which are represented in Figure 4. These enteroendocrine incretin hormones are released from the gut in response to intraluminal carbohydrates [34] and they act as important regulators of post-prandial glycemic control. More specifically, they are involved in several beneficial pancreatic effects, including stimulation of insulin secretion and insulin gene expression, promoting β-cell survival, improving β cell glucose sensitivity and decreasing glucagon secretion. In addition, incretin hormones not only target pancreatic islet cells, but they also possess numerous extra-pancreatic actions that impart positive effects in terms of slowing the gastric emptying and reduction of food intake and weight loss [35].

In the metabolically disturbed state, T2DM subjects show a lacking glucose lowering response mediated by incretin hormone [36]. This is due to reduced activity or desensitization of the GIP-receptors and reduced postprandial circulating level of GLP-1 [37,38]. However, restoring GIP efficacy in T2DM patients is possible: preclinical and clinical studies show that this can be achieved with the enhancement of hyperglycemia due to drugs [39,40] or weight loss [41]. Regarding the glucose dependent insulinotropic hormone, patients treated with GLP-1 for six weeks exhibit an improved insulin-sensitivity and reduced levels of glycated hemoglobin HbA_1c_ [42].

The incretin hormones have indeed a great potential for the treatment of diabetes, but the extremely short biological half-life of these peptides, due to efficient enzymatic degradation by Dipeptidyl Peptidase (DPP)-4 and subsequent renal filtration, severely limits therapeutic applicability [43].

### 2.2. Targeting the Incretin System

#### 2.2.1. Dual Agonists

Although the currently approved GLP-1 receptor (GLP-1R) agonists (Exenatide and Liraglutide) lead to important metabolic improvements, long-term glucose control is still not perfect and reduction in adiposity remains far below from desired. Increasing the dose to gain greater efficacy is not a practicable option for most patients because GLP-1R agonists are source of significant gastrointestinal side effects (i.e., nausea and vomiting) [44]. Therefore, combination therapy appears to be the preferred path to enhance efficacy while maintaining an appropriate tolerability and safety profile.

The design for a dual drug clearly focuses on compounds capable of activating both the predominant endogenous incretins, GLP-1 and GIP. Furthermore, the absence of adverse cardiovascular side effects or neuropsychological complications increases the interest in incretin-based drugs. The design process starts from the observation that GLP-1 and GIP share a high degree of sequence similarity, as shown in Figure 4, which can be modified in order to obtain potent and balanced co-agonism. More specifically this was achieved with the substitution of residues Glu^3^ and Lys^16^ of GIP sequence with the obtained peptides having almost no in vitro glucagon activity. Furthermore, the modifications could include substitution with Lys^40^ that allows site-specific lipidation or PEGylation of the peptide in order to avoid DPP-4 inactivation and consequently to permit less frequent administrations [45].

Several in vivo studies show promising results in terms of decreasing glycated hemoglobin and weight loss in both healthy subjects and patients with T2DM [46].

### 2.3. Targeting the Incretin/Glucagon Systems

#### 2.3.1. Dual Agonists

The design of GLP-1 and glucagon dual agonist has been explored, as well.

Glucagon is a hormone, which participates with insulin in glycemic homeostasis and, while remaining unclear whether low glucose levels directly stimulate glucagon release, it is a fact that T2DM patients suffer from hyperglucagonemia [47], an excess of glucagon secretion, possibly as a result of alpha cell insulin resistance.

Glucagon can be utilized therapeutically as a satiety factor, which also increases energy expenditure and weight loss [48]. Since the incretin hormones and glucagon have some overlapping functions, their combined use could lead to synergistic effects on diabetes and related metabolic diseases [49].

Nevertheless, several studies [50] also verified that acute co-infusion of low doses of GLP-1 and glucagon significantly reduced food intake and increased energy expenditure and this effect was achieved with peptide infused alone as well.

In addition, they have similar peptide sequences at the N-terminal region that allow the development of single-sequence multi-receptor agonists.

**Figure 5:**
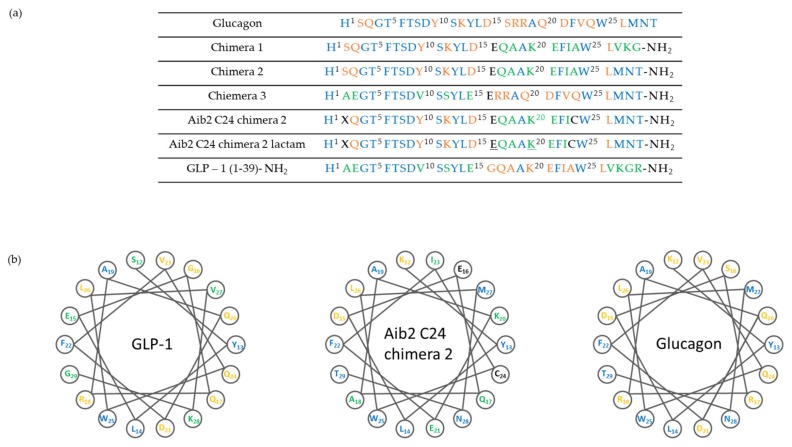
Structure of glucagon, GLP-1 and chimeric peptides. (**a**) Amino acid sequence and structure of glucagon and GLP-1 chimera. Underlined residues indicate site of lactam formation. (**b**) Helical wheel representation of glucagon, GLP-1 and chimeric peptide showing residues 12–29.

Based on the above findings, investigation of compounds simultaneously targeting the GLP-1R and glucagon receptor (GcgR) have been carried out. The research groups of Day et al. [51] and Pocai et al. [52] have first reported peptides that act as such dual drugs and some representative peptides are shown in Figure 5. In preclinical studies, they showed that the treatment with these co-agonists induces superior weight loss and lipid lowering, without causing hyperglycemia or any other adverse effects in diet-induced obese (DIO) mice.

Clearly, the pharmacokinetic profile of these peptides had to be investigated in order to optimize their and co-agonist analogs were rationally designed to possess a spectrum of relative activity ratios at GLP-1R and GcgR. This study [53] revealed that derivatives endowed with comparable activity at each receptor provide optimal weight and blood glucose lowering properties.

The relevance of the results reached with this approach stimulated big Pharma companies such as Sanofi-Aventis and Eli Lilly to invest further in the field. Nevertheless, the risk of unwanted effects of these co-agonists must be carefully assessed, particularly the long-term ones and those affecting cardiovascular health.

**Figure 6:**
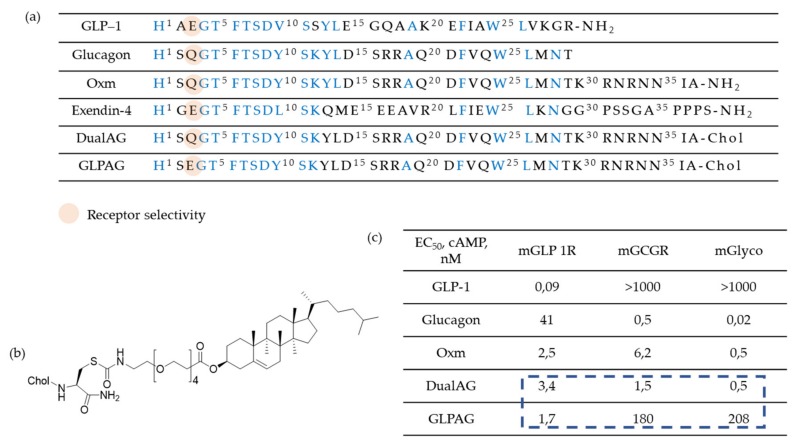
(**a**) Sequence alignment and receptor agonist potencies of Oxyntomodulin (OXM) and related peptides. Conserved residues are highlighted: (**b**) Structure of a Glucagon-Like Peptide 1/Glucagon Receptor Dual Agonist and (**c**) in vitro receptor agonist potencies (cAMP release) against mGLP1R and mGCGR and ED_50_ (nM) in the *ex vivo* mouse liver glycogenolysis assay (mGlyco).

Researchers are also working on oxyntomodulin mimetics. Oxyntomodulin (OXM) is a gut hormone that, similarly to GLP-1, is secreted in response to feeding. Its biological effects are attributed to a dual activation of GLP-1R and GcgR. However, OXM is rapidly inactivated (around 12 minutes) in plasma by DPP-4 which cleavages the first two N-terminal amino acids of the peptide. Therefore, OXM mimetics should inevitably involve a modified N-terminus in order to protect the compound against DPP-4 inactivation and possibly to avoid decreasing of the efficacy. On these bases, investigation of OXM-analogues has been performed [54] to improve half-life. For example, the strategy proposed by Pocai [52] was to introduce a D-Ser^2^ (S) substitution, while an enhanced metabolic stability was achieved through a cholesterol moiety (chol) at the C-terminus of these peptides. Furthermore, the polyethylene glycol spacer minimizes the loss in agonist potency because of plasma protein/lipid binding (Figure 6). Finally, the treatment with these compounds induced similar improvements in glycemic control compared to selective GLP-1R agonists and additionally it has been shown to decrease bodyweight and food intake.

#### 2.3.2. Triagonists

Further studies have investigated engineered peptides to combine the beneficial effects of the incretin hormones and glucagon. Interestingly, both incretin peptides and glucagon are substantially increased following bariatric surgery [55,56], but unfortunately similar benefits cannot be achieved with current pharmaceutical options.

**Figure 7:**
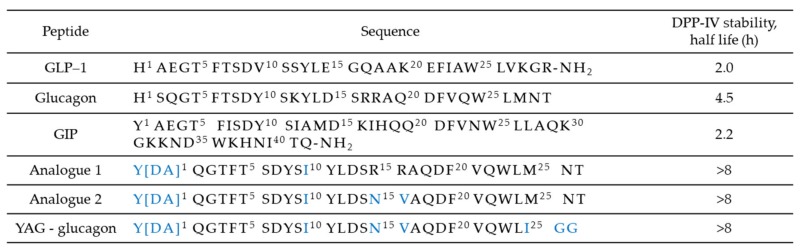
Amino acid sequences of glucagon, the incretin hormones, and triagonist.

All three of these gastrointestinal hormones share similar N-terminal sequences, simplifying the design of single-sequence multi-receptors agonists. A key factor that has to be considered is the relative potency of the compound towards each receptor. Triagonists can be designed in order to achieve a balanced agonism or with an agonism ratio that favors one receptor over the others in function of the desired effects. For example, YAG-glucagon, a glucagon-derived triagonist [57], has reported to have no effect on body weight while significantly improved glycaemia in high fat fed mice due to unbalanced agonism and submaximal potency. The two analogues, namely analogue 1 and analogue 2, shown in Figure 7, were also designed by modification of the amino acid sequence of human glucagon by substituting key amino acids with those that are known to be important in the biological function of GIP, as shown in Figure 4. Balanced triagonists were investigated as well, and recently MAR_423_, developed by Novo Nordisk, entered initial Phase I testing.

### 2.4. Targeting Not Only the Incretin System

**Figure 8:**
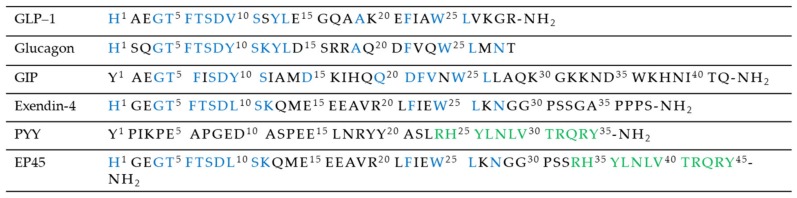
Sequence alignment of peptides Y2, EP45, glucagon, and incretin hormones.

Recently, there is a significant interest towards modified drugs designed to possess the ability to modulate the incretin signaling combined with other system involved in diabetes comorbidities. Literature also reported new strategies in order to lower glycaemia while simultaneously suppressing appetite, such as peptides that act like GLP-1 and other gastrointestinal hormones that participate in the hypothalamic control of appetite. An example could be EP45 (Figure 8), which contains amino acid sequence motifs present within the blood glucose-lowering agent exendin-4 (Ex-4), a GLP-1 ligand, and the appetite-suppressing agent peptide YY (3-36) (PYY(3–36)) [58]. Another example could be a GLP-1/xenin fusion hybrid that incorporates the key amino acid sequences of the same GLP-1 ligand previously mentioned, Ex-4, and a xenin mimetics, xenin-8-Gln, linked via an 8-amino-3,6-dioxaoctanoic acid group [59].

## 3. Targeting Other Systems

### 3.1. SGLT-1/SGLT-2 Inhibitors

Sodium-glucose co-transporter-2 (SGLT-2) is responsible for major glucose reabsorption in renal proximal tubules (around 80–90%) [60,61]. Since its inhibition leads to a reduction in blood glucose level, there is a potential use in the treatment of T2DM. A possible treatment regimen combines SGLT-2 inhibitors plus GLP-1R agonists [62]. These combined inhibitors improve glycaemia control independent of insulin secretion with a low risk of hypoglycemia by decreasing renal glucose reabsorption and increasing urinary glucose excretion [63].

**Figure 9:**
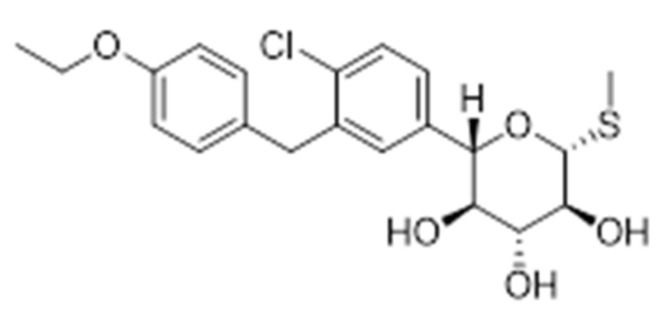
Structure of Sotaglifozin, a Dual SGLT1/SGLT2 Inhibitor.

Due to the beneficial effects of inhibiting SGLT-2, a novel strategy involves the development of dual SGLT-1/SGLT-2 inhibitors. SGLT-1 is the major transporter for glucose absorption in intestine and is also expressed in renal proximal tube. These dual drugs aim to reduce glucose absorption in the gastrointestinal tract, due to the SGLT-1 inhibition, and to reduce renal glucose reabsorption via the inhibition of both transporters. Interesting literature results reported that Sotaglifozin (LX4211), depicted in Figure 9, which inhibits both SGLT-1/SGLT-2 increases glucagon-like peptide-1 and peptide YY levels [64] and demonstrates that this approach minimizes the risk of hypoglycemia and body weight gain, two main challenging clinical goals in a glucose-lowering treatment. Sotagliflozin is now under development by Lexicon pharmaceuticals for the treatment of T1DM and T2DM.

### 3.2. AR/PTP1B Dual Inhibitors

**Figure 10:**
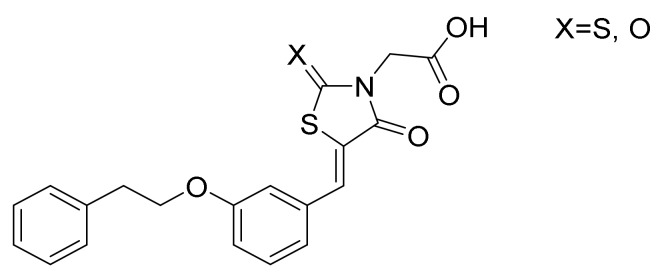
General structure of aldose reductase (AR)/protein tyrosine phosphatase 1B (PTP1B) inhibitors.

Novel molecular targets are also investigated like aldose reductase (AR) and protein tyrosine phosphatase 1B (PTP1B), two key enzymes involved in different events which are critical for the onset and progression of T2DM and related comorbidities. The former is a key enzyme in the polyol pathway which could induce an excessive accumulation of intracellular reactive oxygen species (ROS) in several tissues, such as heart, vasculature, eyes and kidneys, which could be implied in many diabetic complications. Regarding PTP1B, this enzyme has been implicated in the negative regulation of both insulin and leptin. Furthermore, it may be also involved in insulin-resistance and obesity. The research group of Ottanà [65] developed several 4-thiazolidinone derivatives as potential inhibitors of both AR and PTB1B (Figure 10). These compounds will be further optimized in order to balance the inhibitory profile since they show a much more potent activity toward human AR.

Since disease management also takes care of cardiovascular risk factors such as arterial hypertension, alteration in triglycerides, cholesterol levels, and increased production of uric acid, which usually coexist in the same patient, other current therapeutic strategies are targeting the entire spectrum of these dysfunctions and their relatively regulatory pathways. In this context, a noticeable trend is focused on the design and synthesis of dual peroxisome proliferation activated receptors (PPARs) agonists.

## 4. PPARs-Based Therapies

### 4.1. Overview

PPARs comprise a group of nuclear receptor proteins, codified by different genes that have three subtypes with a specific tissue distribution: PPAR-α, PPAR-δ (also known as PPAR-β), and PPAR-γ. PPAR-α is highly expressed in tissues with high fatty acids oxidation such as liver, kidney, heart muscle, and vascular endothelial cells since its activation promotes lipid metabolism and consequently increases plasma HDL-Cholesterol (HDL-C) levels. Moreover, several studies have demonstrated anti-atherogenic and anti-inflammatory activities of PPAR-α which can be attributed to the inhibition of several inflammatory mediators and adhesion molecules [66,67]. The reducing of lipotoxicity and inflammatory mediators in long-term treatment with PPAR-α agonist have shown an improvement in cardiac performances in diabetic patients and a reduction in diabetes-associated cardiovascular risk factors [68].

PPAR-δ has a broad expression pattern and plays important roles in the regulation of proliferation and differentiation of several cell types, including adipose cells. Studies on PPAR-δ show also its implication in the inflammatory status of the macrophage, which suggests that its modulation has the potential to attenuate inflammation and slow down the progression of atherogenesis [69]. PPAR-γ is expressed mainly in the adipose tissue, intestinal cells, and in mononuclear leukocytes, where it is involved in adipocyte proliferation and differentiation. Several studies show that PPAR-γ ligands have pleiotropic effects in cardiovascular complications [70].

PPARs can regulate gene transcription by binding to specific DNA response elements upon ligand activation and heterodimerization with the 9-cis retinoic acid receptor (RXR). The different subtypes can be activated by different endogenous ligands like free fatty acids, eicosanoids, and Vitamin B3, but they are also several marketed drugs, such as fibrates, a hypolipidemic class which acts through PPAR-α, and thiazolidinediones, antidiabetic agents which can activate PPAR-γ.

The design of dual PPARs agonists are currently under development in order to produce synergistic anti diabetic (reduce hyperglycemia and hyperlipidemia) and cardioprotective effects that could be more efficient than the treatment with selective agonists [71].

### 4.2. Dual Agonists

The most promising efforts were made in the development of PPAR-α/γ dual agonist which revealed potent therapeutic effects for DM, cardioprotective effects, and dyslipidemia.

**Figure 11:**
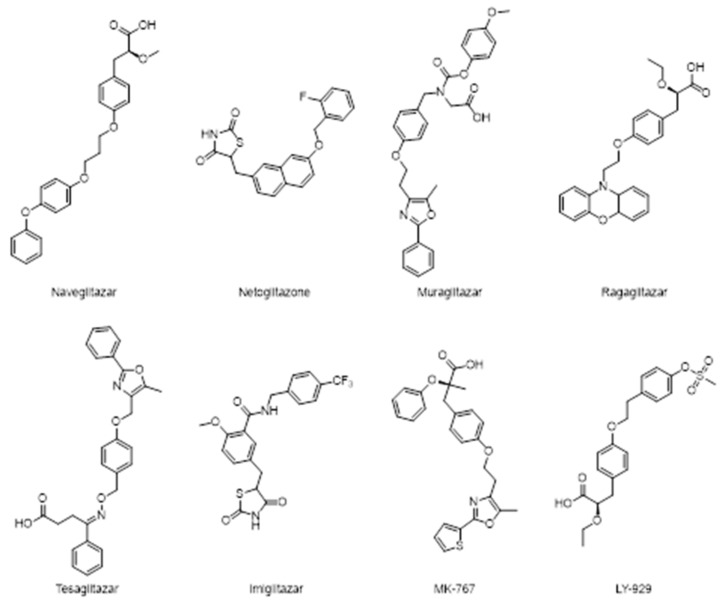
Structures of several peroxisome proliferation activated receptor (PPAR)-α/γ dual agonists.

Figure 11 reports some recently investigated PPAR-α/γ dual agonists: naveglitazar, netoglitazone, muraglitazar, ragaglitazar, tesaglitazar, imiglitazar, MK-767, and LY-929.

Clinical trials with dual PPAR-α/γ agonists demonstrate reduction in triglycerides concentration, an increase in cardioprotective HDL-C level with consequently improvements in insulin sensitivity [72].

Evidences reported in literature demonstrated the positive effects of the dual agonist ragaglitazar in decreasing cardiovascular risk factors by inducing an efficient hypotensive effect in spontaneously hypertensive rats and in the improvement of the endothelial function in Zucker diabetic fatty rats [73].

Ragaglitazar was also reported to have some carcinogenic effects in rodent but there are not enough available data that support this, as well as the effects have been never observed in human. MK-767 has slightly less activity on PPAR-γ when it compared with pioglitazone. MK-767 effectively normalizes hyperglycemia and hyperinsulinemia in the diabetic ab/ab mouse model. In healthy human subjects, it still reduces triglyceride, FFA, LDL, VLDL, and fasting plasma glucose level. Furthermore, it increases adiponectin levels in healthy subjects [71].

The clinical program of MK-767 was developed by Merck in collaboration with Kyorin Pharmaceutical Co., but it was halted at phase III, in a long-term safety assessment program, due to the identification of a rare form of malignant tumor in mice.

Unexpectedly, the developments of other dual PPARs agonist such as tesaglitazar has been withdrawn in phase III clinical trials because it may cause an increase in fibro sarcoma formation.

Muraglitazar has been discontinued as well because, despite it is responsible for an increase in HDL-C levels, and a decrease in total cholesterol, apolipoprotein B, triglycerides and HbA_1c_, this agent is associated with an increased risk of adverse cardiovascular events and heart failure.

These unsatisfactory results led to the investigation of the agonist ratio in order to avoid the exacerbation of adverse effects and the gained lines of evidence show that supra activation of PPAR- α or γ may be associated with diabetes comorbidities such as renal dysfunction, fluid retention, heart failure, and carcinogenesis.

Tesaglitazar and muraglitazar also lack of balance in binding affinity: the former has high affinity towards PPAR-γ while the latter is more active on PPAR-α.

Hence, one of the challenges is the design of a dual agonist with balanced PPAR-α/γ activity and good safety profile.

**Figure 12:**
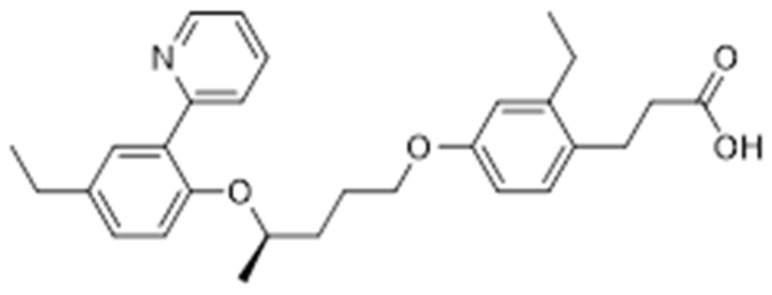
Chemical structure of a PPAR-γ/δ agonist.

Further studies have been looking for combining the potential effects of PPAR-α/δ and PPAR-γ/δ, which may show interesting proprieties like the dual activation of PPAR-α/γ. For example, (R)-3-{2-ethyl-4-[3-(4-ethyl-2-pyridin-2-yl-phenoxy)-butoxy]-phenyl}propionic acid (Figure 12), a PPAR-γ/δ agonist, has been shown to lower the glucose level inducing less weight gain than rosiglitazone [74].

Furthermore, these agents possibly avoid weight gain, an adverse effect associated with thiazolidinediones treatment, but still further studies must be done to investigate the efficacy and safety of these compounds.

### 4.3. Pan Agonists

**Figure 13:**
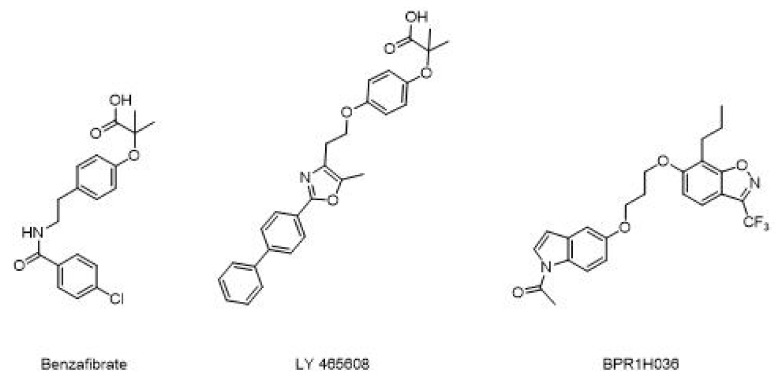
Different example of PPARs pan agonist.

On the other hand, development of pan agonists, which can act on more than two targets, is current under investigation. Pharmacodynamic tests on benzafibrate demonstrate that this lipid lowering drug acts on all the three PPARs subtypes and induces lower LDL cholesterol and triglyceride levels, while increasing HDL. Additional studies show that in patients with impaired glucose balance, benzafibrate may delay progress of diabetes by improving insulin sensitivity, inhibiting atherosclerosis, and preventing ischemic heart injury [75]. Furthermore, these agents are also expected to avoid weight gain. Benzafibrate analogues (Figure 13) such as LY 465608 and BPR1H036 are under investigation as novel PPARs pan agonist [76]. However, in vitro and in vivo studies have to be performed to collect more clinical evidence of their efficacy.

## 5. Herbal Medicines Approach

Like multi-target ligands, phytocomplex exploits the “herbal shotgun” effect where multiple constituents interact with different targets. The multi-targets approach is in contrast with the “silver bullet” one that refers to the effect of a single substance acting at a single target. Several plant extracts were tested to corroborate their synergistic and multifactorial effects towards DM [77]. Here are represented a few of those. Among the herbal products that are not mentioned in this review, gurmar (Gymnema sylvestre), ivy gourd (Coccinia indica), cinnamon (Cinnamomum cassia), psyllium (Plantago ovata), and garlic (Allium sativum) deserve a particular mention for their therapeutic effects on T2DM [78].

### 5.1. Momordica Charantia

Momordica charantia, also known as Bitter Melon (BM), is a plant indigenous to tropical and subtropical regions including South America, Asia, India and East Africa and has been traditionally used in Asian phytotherapic treatment of T2DM. Several studies were carried out in order to investigate the different pharmacological mechanisms of actions [79], but they are not completely elucidated yet. The increased expression of PPAR-γ and reduced leptin expression in white adipose tissues, as well as the promotion of GLUT4 expression in skeletal muscles in high-fructose-fed rats are among the proposed mechanisms. Regardless the mechanisms, its extracts improved insulin sensitivity, glucose tolerance and insulin signaling pathway in high fat-fed rats [80]. Furthermore, other pharmacological effects include the suppression of postprandial hyperglycaemia [81], while cucurbitane-type triterpene glycosides and trehalose isolated from the seeds inhibited alpha-glucosidase enzyme activities [82].

### 5.2. Panax Ginseng

The activity as anti-diabetic agents of different ginseng species seems due to its saponin and polysaccharide constituents which might be involved in the double activation of AMP-activated protein kinase (AMPK) and PPAR-γ [83]. They act with different mechanisms including the stimulation of insulin biosynthesis and its secretion. Furthermore, consumption of ginseng increases insulin-regulated receptor (GLUT-4) in skeletal muscle and in liver in obese mice and increases lipoprotein lipase (LPL) and PPAR-γ in adipose tissues [84].

### 5.3. Trigonella Foenum-Graecum

Historically Trigonella foenum-graecum, fenugreek, was used for a variety of health conditions, including diabetes. Studies report that it can inhibit carbohydrate metabolic enzymes [77] leading to hypoglycemic effects. Furthermore, its seeds can decrease glucose-6-phosphatase and fructose-1,6-bisphosphatase in liver and kidney.

### 5.4. Scutellariae Radix

Scutellariae Radix (SR) is a dry root of Scutelleria baicalensis Georgi which has been used to treat different types of diseases. Its biological activities include anti-inflammation, anti-cancer, and anti-oxidation effects. The pharmacologically active components are flavonoids, such as baicalin, wogonoside, baicalein, and wogonin and their main beneficial effects involve the improvement in insulin resistance and the suppression of gluconeogenesis.

### 5.5. Coptidis Rhizoma

Coptidis Rhizoma (CR) is a dried rhizome of Coptis chinensis. It mainly contains alkaloids, such as berberine, coptisine, and palmatine, as pharmacologically active components. Recently studies have shown that CR has anti-bacterial, anti-cancer activities. Moreover, berberine was found to be able to lower blood glucose and promoting the secretion of insulin.

Therefore, SR and CR could alleviate inflammation, insulin resistance, hyperglycemia and hyperlipemia, which are mostly contribute to diabetes disease. The combined extracts of SR and CR (1:1 ratio) have been used in therapies of traditional Chinese medicine to obtain a synergistic effect to treat T2DM. However, their compatibility mechanism remains unknown, but the metabolomics and MAPK/PI3K/Akt signaling pathway helped to unraveled it.

## 6. Conclusions and Future Directions

As mentioned in the Introduction section, since diabetes has a multifactorial pathological nature, it comes as no surprise that concurrent interactions of more than one potential modulator appear to have promise for future treatments. This may be achieved with a new approach, more specifically through the development of multi modal compounds. Unfortunately, the clinical development of some multifunctional ligands has been discontinued because of their undesirable side effects, maybe due to their imbalanced and/or supra-therapeutic activity. Given this, the potential promise of compounds able to modulate the activity of multiple targets still requires detailed investigations.

Future advances in the understanding of the genetics base and of the signaling pathways which characterize the disease, coupled with their therapeutic applications, should lead to an expansion of new treatments, like personalized medicine [85,86], that could exploit new clinically available multi-target drugs. Tailoring medical therapies to the patient’s biological characteristics may help to optimize disease treatment, thereby improving overall prognosis and decreasing comorbidities’ risk.
